# Successful diagnosis and treatment of early splenic ectopic pregnancy: A case report

**DOI:** 10.1097/MD.0000000000010466

**Published:** 2018-04-27

**Authors:** Lan Wu, Xiaoqin Jiang, Juan Ni

**Affiliations:** Key Laboratory of Birth Defects and Related Diseases of Women and Children (Sichuan University), Ministry of Education, Chengdu, Sichuan Province, China.

**Keywords:** diagnosis and treatment, ectopic pregnancy, splenic ectopic pregnancy

## Abstract

**Introduction:**

Splenic ectopic pregnancy (SEP), a special abdominal pregnancy, is extremely rare but carries a high risk of potentially uncontrollable, life-threatening intraperitoneal bleeding at early gestation, which is equivalent to the spontaneous rupture of the spleen. Therefore, early diagnosis of SEP is crucial and may avoid life-threatening situation.

**Case presentation:**

A 29-year-old G3P2 woman presented with 50 days of amenorrhea and positive serum β-human gonadotropin (β-HCG) was enrolled into the hospital due to the absence of gestational sac located in the uterine cavity. A pan-abdominal ultrasound scan revealed a 2.6 cm ×1.6 cm hyperechoic mass inferior to the spleen with color Doppler signal surrounding and 0.9 cm anechoic inside. The gynecologist found the gestational sac was located in the dorsal pole of the spleen through the exploratory laparoscopy. Total splenectomy was performed uneventfully to avoid the hemorrhage shock. The patient discharged with no complications and normal 1-month follow-up.

**Conclusion:**

It highlights that fully understanding of the knowledge about abdominal pregnancy, especially splenic pregnancy, and early imaging study with ultrasonography could reduce or avoid the misdiagnosis and miss-diagnosis of SEP.

## 1 Introduction

Ectopic pregnancy is defined as the implantation of a fertilized ovum outside of the uterine cavity with the estimated incidence of 19.7/1000.^[1]^ While 95.5% of the ectopic pregnancies are in the fallopian tube, only 1.3% are abdominal in location.^[2]^ Although a variety of extra pelvic organs such as the liver, omentum, and intestines have been described in the past, reports of primary pregnancy in the spleen are very rare. The spleen cannot accommodate placental attachment and therefore patients often suffer rupture and life-threatening hemoperitoneum requiring emergency surgical intervention.^[3]^ Early diagnosis is crucial, as delay can result in loss of fertility or even maternal mortality.^[4]^ We here present a case of clinically asymptomatic splenic pregnancy which was diagnosed and treated during the early stage. Informed consent was obtained from the patient for publication.

## 2 Case report

**Figure 1:**
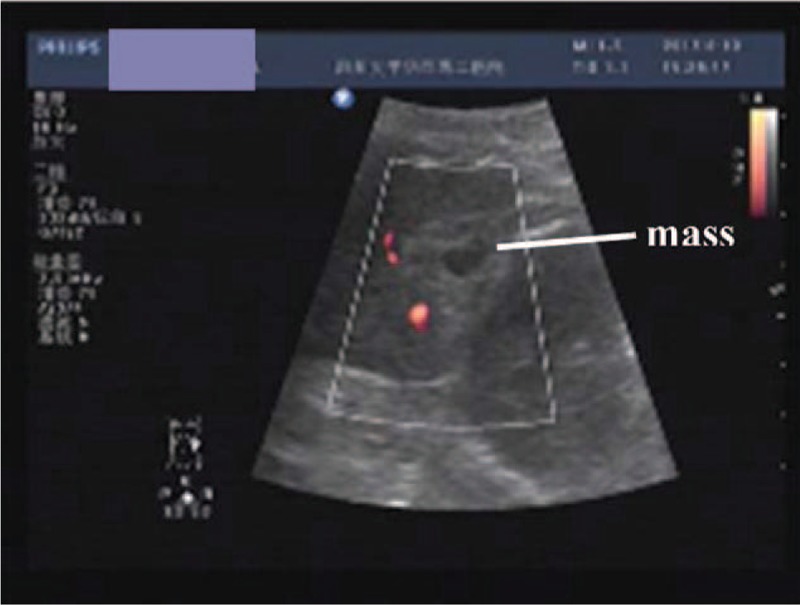
Abdominal ultrasound showing the presence of a well-defined heterogeneous a lightly hyperechogenic mass with anechoic area (0.9 cm in diameter) inside at the lower splenic pole.

A healthy 29-year-old woman, G_3_P_2_, presented with chief complaint of amenorrhea for 50 days. She had regular menstrual periods in the past. The cycle of menses was usually regular (28 days) since menarche, and her last menstruation was on June 24, 2017. She had an episode of spotty vaginal bleeding of 3 days before her due period date. She presented to emergency room at outside hospital with mild right upper quadrant pain on 39th day of amenorrhea. Physical examination was otherwise unremarkable. She was treated with intravenous antibiotics for 3 days with complete relief of pain. Neither blood β-human gonadotropin test nor pelvic ultrasound study was performed at that time. The patient then presented to our hospital with breast discomfort and positive urine β-human gonadotropin on 49th day of amenorrhea. Her serumβ-human gonadotropin (β-HCG) level was 16669 IU/L on the same day. Transvaginal sonography showed no gestational sac located in the uterine cavity; no mass in the adnexal area and no fluid within the pelvic cavity. A pan-abdominal ultrasound scan was then performed and revealed a 2.6 cm ×1.6 cm hyperechoic mass inferior to the spleen with color Doppler signal surrounding and 0.9 cm anechoic inside (Fig. 1).

**Figure 2:**
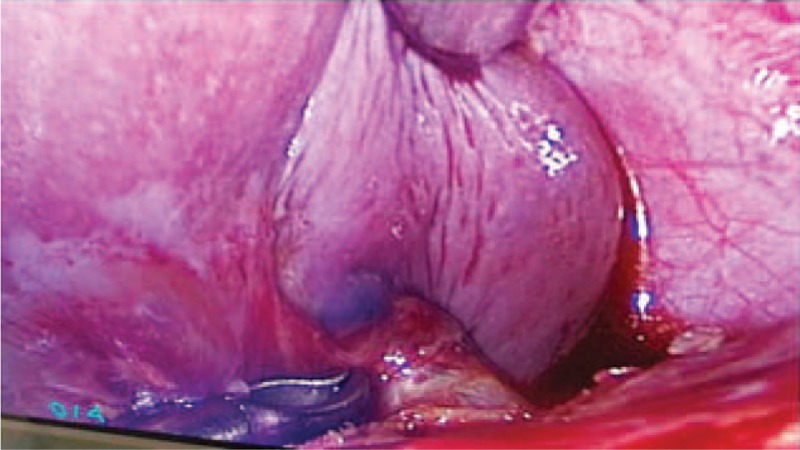
A purple blue sac-like nodule (3 cm in diameter) in the dorsal pole of the spleen was checked by laparoscopy.

**Figure 3:**
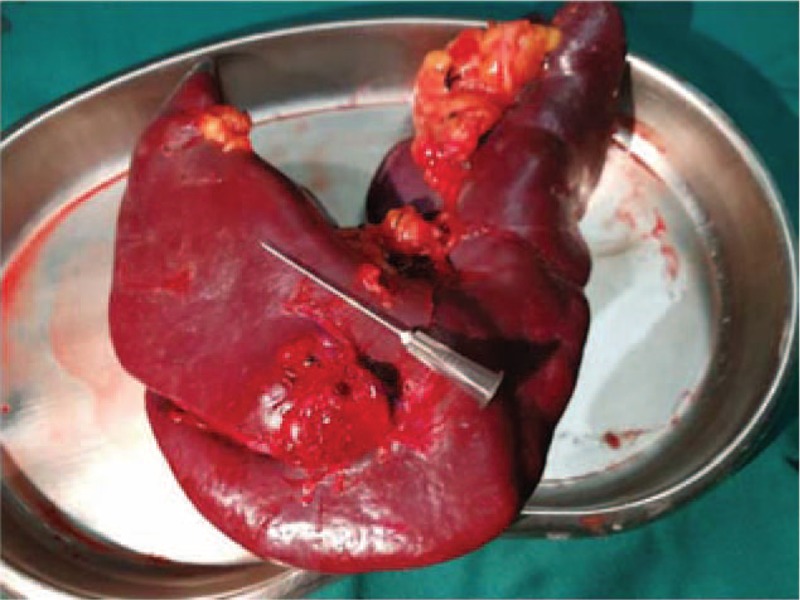
Excised spleen (10.0 × 8.0 × 5.0 cm) and the chorionic villi (3.0 × 3.0 cm).

These abdominal ultrasonography findings and elevated serum β-human gonadotropin (β-HCG) level led to a suspected diagnosis of splenic ectopic pregnancy. Exploratory laparoscopy was then performed with general anesthesia. The uterus and both ovaries and fallopian tubes were normal in appearance, showing no signs of ectopic pelvic pregnancy. Further exploration revealed that a spleen size of 10 cm × 8 cm × 5 cm, and a purple bluish nodule with diameter of approximately 3 cm in the dorsal pole of the spleen (Fig. 2). Total splenectomy was performed uneventfully. Gross examination of the spleen revealed an identifiable chorionic villi invading the splenic parenchyma with a size of approximately 3.0 cm × 3.0 cm (Fig. 3). Frozen section analysis confirmed chorionic villi. A histopathologic diagnosis of primary splenic ectopic pregnancy was then made.

**Figure 4:**
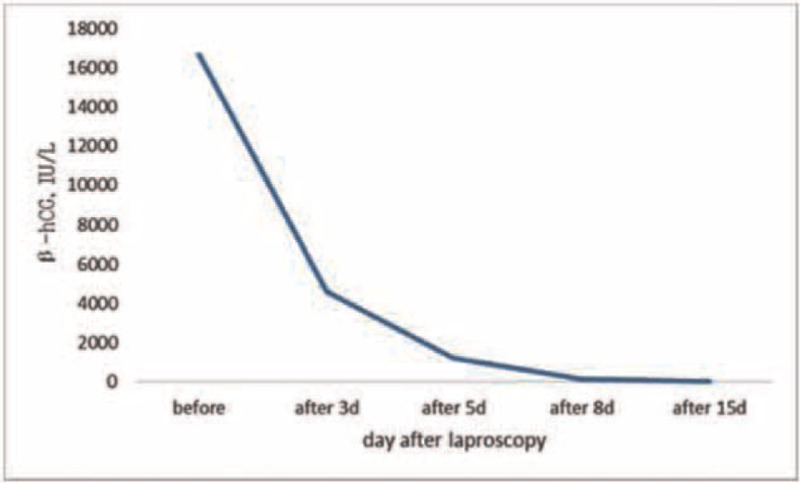
Changes in serum β-human chorionic gonadotropin (β-hCG) concentration after splenectomy. β-hCG = β-human chorionic gonadotropin.

The patient recovered uneventfully and was followed-up with plasma β-HCG concentration and transvaginal ultrasound. On postoperative day # 3, serum β-HCG concentration was 4618 IU/L, decreased to 1209 IU/L, 134.9 IU/L and 13.7 IU/ L on postoperative day #5, #8, and # 15, respectively (Fig. 4). An abdominal ultrasound examination was performed 1 month after surgery and revealed no continued splenic pregnancy.

## 3 Discussion

Splenic ectopic pregnancy (SEP), a very rare type of ectopic pregnancy, carries a high risk of potentially uncontrollable, life-threatening intraperitoneal bleeding at early gestation.^[2,5–11]^ Therefore, early diagnosis of SEP is critical, and may avoid life-threatening situation. Early recognition and diagnosis is usually overlooked when clinically asymptomatic. A review of the international literature^[12]^ showed only 17 reported cases of splenic ectopic pregnancy. Of the cases identified, nearly all patients presented with hemoperitoneum or intraabdominal bleeding. Only 1 patient was asymptomatic and stable when the diagnosis was made incidentally by abdominal CT scan. We reported the second successful diagnosis and treatment of early splenic ectopic pregnancy.

Early clinical symptoms of splenic pregnancy are not typical and can easily lead to misdiagnosis or even missed diagnosis. Splenic pregnancy would cause rupture with massive hemoperitoneum, and usually constitute an urgent medical situation if the pregnancy was left untreated. Therefore, early recognition and diagnosis and timely treatment of splenic pregnancy are very important.

To help early diagnosis, a few things need to be emphasized. Firstly, fully understanding of abdominal pregnancy, especially rare types as splenic pregnancy, and a thorough history taking and physical examination can help identify possible abdominal pregnancy. Gao et al^[6]^ reported that the reason for the missed diagnosis at the first visit in their case may had been due to lack of knowledge about abdominal pregnancy, especially splenic pregnancy. Therefore, clinician should always stay vigilant of the possibility of rare types of abdominal pregnancy such as splenic pregnancy and an interdisciplinary investigation may be warranted if index of suspicion was high.

Second, we had emphasized the value of abdominal ultrasonography or abdominal CT in the early diagnosis of a splenic pregnancy. Ultrasonography is probably the most important tool in the diagnosis of an ectopic pregnancy. Our case was a successful example of early diagnosis and treatment of splenic pregnancy. Interestingly, in our case, the slight abdominal pain complained by the patient complete relieved after the antibiotics infusion, even though the gestational sac was relatively large (2.6 cm × 1.6 cm). The key to the successful diagnosis was the utilization of pan-abdominal ultrasonography. None of the cases published had been diagnosed with an ultrasound prior to treatment. Our case was the first one. Klang et al^[13]^ reported that the diagnosis was made by abdominal CT before treatment. The movement of the patient, especially the exercise increasing the intra-abdominal pressure, increases the risk of gestational sac rupture. The ultrasound image could be obtained easily in the consulting room in order to minimize the movement of the patient. Ultrasonography should be considered the standard imaging tool. If a screening abdominal ultrasound was performed, pregnant sac-like echo image may be seen around the spleen.

Patients with splenic pregnancy might have atypical abdominal pain, and sometimes this mild pain was easily overlooked before splenic rupture occurred. Some patients showed sudden onset of the left upper or full abdominal pain, if bleeding had happened. In our case, the patient presented with complaints of brief intermittent left upper abdominal pain which was attributed to other etiology. For patients with suspected ectopic pregnancy, a thorough interview and physical examination should be performed, focusing on the features of the abdominal pain such as location, quality, exacerbating/alleviating factors, etc., which may help identify the possible site of ectopic pregnancy. Other than the common sites of ectopic pregnancy, we should also consider the possibility of a pregnancy at abdominal viscera such as bowel, omentum, liver, and spleen. If there was a history of atypical abdominal pain, possibility of an ectopic abdominal pregnancy should be considered, in particular, when ectopic pregnancy was suspected but no gestational sac was found in the pelvic.

Finally, splenic pregnancy is a potentially life-threatening condition. If splenic pregnancy was suspected by abdominal ultrasound, an emergent laparoscopic examination should be strongly recommended, and the entire abdominal cavity must be evaluated if possible. Laparoscopic examination seemed to be safe and effective, enabling a reliable and early diagnosis. In our case, the entire abdominal cavity was evaluated with no abnormalities in the fallopian tubes or the uterus but in the spleen. Splenectomy was performed which eliminated the risk of rupture of splenic pregnancy and the possible requirement of blood transfusion.

In summary, we reported a case of clinically asymptomatic splenic pregnancy that was successfully diagnosed and treated. With substantially high β-hCG level and transvaginal ultrasonography revealing no intrauterine gestational sac, the clinician should consider the possibility of an ectopic abdominal pregnancy including splenic/liver/omentum pregnancy and a pan-abdominal ultrasonography should be done to avoid misdiagnosis and missed diagnosis. If the splenic pregnancy was suspected by abdominal ultrasound, an emergent laparoscopic exploration must be performed for definitive diagnosis and possible treatment, if feasible. Early successful diagnosis of this rare form of gestation is of considerable importance because of the risk of a life-threatening intraperitoneal hemorrhage.

## Author contributions

**Data curation:** Juan Ni.

**Project administration:** Xiaoqin Jiang.

**Writing – original draft:** Lan Wu, Xiaoqin Jiang.

**Writing – review & editing:** Xiaoqin Jiang.
