# Successful diagnosis and treatment of early splenic ectopic pregnancy: A case report: Erratum

**DOI:** 10.1097/MD.0000000000012141

**Published:** 2018-08-24

In the article, “Successful diagnosis and treatment of early splenic ectopic pregnancy: A case report”,^[1]^ which appeared in Volume 97, Issue 17 of *Medicine*, the affiliations for both authors should be ^a^ West China Second University Hospital and ^b^ Key Laboratory of Birth Defects and Related Diseases of Women and Children (Sichuan University), Ministry of Education.
